# Uric Acid and Vascular Damage in Essential Hypertension: Role of Insulin Resistance

**DOI:** 10.3390/nu12092509

**Published:** 2020-08-19

**Authors:** Velia Cassano, Daniele Crescibene, Marta Letizia Hribal, Corrado Pelaia, Giuseppe Armentaro, Marcello Magurno, Alfredo Toscani, Sofia Miceli, Francesco Andreozzi, Raffaele Maio, Maria Perticone, Giorgio Sesti, Francesco Perticone, Angela Sciacqua

**Affiliations:** 1 Department of Medical and Surgical Sciences; University Magna Græcia of Catanzaro, 88100 Catanzaro, Italy; velia.cassano@libero.it (V.C.); daniele.crescibene@gmail.com (D.C.); hribal@unicz.it (M.L.H.); pelaia.corrado@gmail.com (C.P.); peppearmentaro@libero.it (G.A.); mmagurno14@gmail.com (M.M.); a.toscani91@gmail.com (A.T.); sofy.miceli@libero.it (S.M.); andreozzif@unicz.it (F.A.); raf_maio@yahoo.it (R.M.); mariaperticone@hotmail.com (M.P.); perticone@unicz.it (F.P.); 2 Department of Clinical and Molecular Medicine, University of Rome-Sapienza, 00161 Rome, Italy; giorgio.sesti@uniroma1.it

**Keywords:** insulin resistance, uric acid, arterial stiffness, atherosclerosis, hypertension

## Abstract

Increased levels of uric acid (UA) have been shown to be correlated with many clinical conditions. Uric acid may adversely affect the insulin signalling pathway inducing insulin resistance (IR). Several studies report the association between arterial stiffness (AS), an early indicator of atherosclerosis, and UA. The purpose of the present study was to evaluate the association between UA and AS, considering the potential role of IR. We enrolled 1114 newly diagnosed, never-treated hypertensive patients. Insulin resistance was assessed by the homeostatic model assessment (HOMA) index. Arterial stiffness was evaluated as the measurement of the carotid–femoral pulse wave velocity (PWV). The study cohort was divided into subgroups, according to increasing tertiles of UA. The mean values of UA were 5.2 ± 1.6 mg/dL in the overall population. Pulse wave velocity was linearly correlated with UA (*p* < 0.0001), HOMA (*p* < 0.0001), high sensitivity C-reactive protein (*p* < 0.0001), systolic blood pressure (*p* < 0.0001) and LDL cholesterol (*p* = 0.005). Uric acid was the strongest predictor of PWV and was associated with the highest risk for increased AS. The interaction analysis showed that the joint effect of increased UA and HOMA was significantly higher than that expected in the absence of interaction under the additive model, indicating that the two biomarkers synergically interacted for promoting vascular damage. Our data showed that UA interacted with IR to increase AS in a large cohort of newly diagnosed, never-treated hypertensive patients.

## 1. Introduction

Uric acid (UA) is mainly synthesized by the hepatic tissue as the end product of purine catabolism and excreted by the kidney; it circulates predominantly in the form of a monovalent sodium salt (urate) [1]. Hyperuricemia (i.e., increased serum UA levels) is caused by either lower secretion, higher synthesis, or a combination of both these defects. It is widely recognized that hyperuricemia is not only a biomarker for several cardio-metabolic disorders, including obesity, dysglycemic conditions, hypertension, and atherosclerotic disease [2,3,4], but also represents an independent predictor of cardiovascular morbidity and mortality as well as of type 2 diabetes mellitus (T2DM) [5,6,7,8,9,10]. Several mechanisms have been proposed to explain these associations. Interestingly, it has been shown that UA promotes oxidative stress and vascular inflammation [11], reducing nitric oxide bioavailability thus favouring endothelial dysfunction [12]. Moreover, UA may directly affect the intracellular insulin signalling pathway, consequently inducing insulin resistance (IR) [13]. All these mechanisms may, in turn, cause subclinical organ damage, and accumulating evidence indicates that higher UA concentrations are associated with arterial stiffness (AS), a marker of vascular damage, and an independent predictor of cardiovascular (CV) events. However, it is not known whether this association persists after adjustment for known CV risk factors, or if UA interacts with other risk factors, such as IR, to promote vascular damage. The purpose of this study was to evaluate the association between circulating UA levels and AS in a large cohort of newly diagnosed, never-treated hypertensive Caucasian individuals, considering the potential role of IR in this context. 

## 2. Material and Methods

### 2.1. Study Population

We recruited 1114 (480 females and 634 males, mean age 48.9 ± 11.5) newly diagnosed hypertensive, never-treated, non-diabetic Caucasian individuals participating in the Catanzaro Metabolic Risk factors (CATAMERI) Study [14]. All patients underwent review of their medical history and physical examination. A complete anthropometrical assessment was carried out with measurement of height, weight, and body mass index (BMI). All subjects had a normal renal function, defined as estimated glomerular filtration rate (e-GFR) > 60 mL/min/1.73 m^2^. None had a positive history or clinical evidence for myocardial infarction, angina, valvular heart disease, hypercholesterolemia, diabetes mellitus, coagulopathies, peripheral vascular disease, or diseases predisposing to Raynaud’s phenomenon or vasculitis. Additional exclusion criteria were post-menopause or hormonal substitutive therapy for women, and positive anamnesis for drug or alcohol abuse, gout, and/or treatment with allopurinol and/or other drugs that may affect UA metabolism. Secondary forms of hypertension were excluded by systematic testing following standard clinical procedures, including measurement of aldosterone and plasma renin activity, Doppler studies of the renal arteries, and/or renal angiography or renal scintigraphy. 

### 2.2. Blood Pressure Measurements

Measurements of clinic blood pressure (BP) were performed after 5 min of rest in the left arm of the supine subjects with an aneroid sphygmomanometer. A minimum three BP records were considered on three different measurements at least two weeks apart. Systolic and diastolic BPs were registered at the first appearance (phase I) and the disappearance (phase V) of Korotkoff tone. Baseline values of BP were calculated from the mean of the last two of the three consecutive recordings performed at three minutes intervals. Subjects with a systolic BP (SBP) > 140 mmHg and/or a diastolic BP (DBP) > 90 mmHg were considered hypertensive in accordance with current guidelines [15]. 

### 2.3. Laboratory Determinations

All laboratory measurements were performed following a 12 h minimum fasting period. Plasma glucose concentrations were measured by the method of glucose oxidation (Beckman Glucose Analyzer II; Beckman Instruments, Milan, Italy). Triglycerides, total, high- (HDL) and low-density lipoprotein (LDL) cholesterol concentrations were evaluated by enzymatic methods (Roche Diagnostics GmbH, Mannheim, Germany). Concentration of plasma insulin was assessed by a chemiluminescence-based assay (Roche Diagnostics). Insulin sensitivity was calculated with the homeostasis model assessment (HOMA) index, computed using the following formula: (fasting insulin (µU/mL) × fasting glucose (mmol/L))/22.5. Serum creatinine was assessed using the Roche Creatinine Plus assay (Hoffman-La Roche, Basel, Switzerland) on a clinical chemistry analyser (Roche/Hitachi Modular Analytics System, P Module). Renal function, evaluated by e-GFR, was calculated according to the equation suggested by the Chronic Kidney Disease Epidemiology (CKD-EPI) Collaboration group [16]. Serum UA levels were measured by the URICASE/POD method on an automated analyser (Boehringer Mannheim, Mannheim, Germany).

### 2.4. Pulse Wave Reflection and Central BP Measurements

All measurements were conducted using a widely validated system (Sphygmocor™; AtCor Medical, Sydney, Australia) based on high-fidelity applanation tonometry (Millar) and appropriate software for the analysis of pressure waves (Sphygmocor™), as previously reported [17]. After a 30 min rest, pressure calibration was acquired with patient in supine condition, through non-invasive, automatic recording of brachial artery BP at the dominant arm, (Dinamap Compact T; Johnson & Johnson Medical Ltd., Newport, UK). Blood pressure was estimated five times at 10 min intervals, and for the calibration, the mean value of the last three measurements was considered. The pulse wave was recorded at the radial artery of the dominant arm with the wrist softly hyperextended, and the measurement was the average of single pressure waves recorded consecutively for eight seconds. Pulse wave recordings were accepted only if the variation of the peak and bottom values of single waves was <5%. The central pressure wave was automatically derived from the radial pressures with a built-in generalized transfer function. Moreover, pressure waves were also measured at the right carotid artery, because it is well known that central augmentation index (AI) may be more precise when obtained from this vascular area [17]. Central pulse waves were also analysed to recognize the time to peak/shoulder of the first (T1) and second (T2) pressure wave components during the systolic phase. The pressure at the peak/shoulder of T1 was defined as outgoing pressure wave height (P1), the pressure at the peak/shoulder of T2 was identified as the reflected pressure wave height (P2), either absolutely or as percent of ejection duration. Augmentation pressure (AP) was identified as difference between P2–P1, and AI as (AP/pulse pressure (PP)) x 100. Aortic pulse wave velocity (PWV) was derived from carotid and femoral pressure waveforms. Carotid to femoral transit time (ΔT) was calculated from the foot-to-foot time difference between carotid and femoral waveforms. The distance between the landmark of the sternal notch and femoral artery was used to estimate the path length between the carotid and femoral arteries (L), and PWV measured as L/ΔT [18]. Carotid–femoral PWV is the gold standard to assess aortic stiffness, and a threshold of >10 m/s has been suggested as a conservative estimate of significant alterations of aortic function in hypertensive patients [19,20]. 

### 2.5. Statistical Analysis

Baseline characteristics are reported as mean ± SD for continuous variables and frequencies and percentages for categorical data. One-way ANOVA and chi-square test were used to test for differences among tertiles of UA concentrations for categorical values and continuous variables, respectively. Linear regression analysis was employed to assess the correlation of PWV with the following covariates: age, BMI, SBP, DBP, HDL and LDL-cholesterol, triglycerides, high sensitivity C reactive protein (hs-CRP), e-GFR, HOMA and serum UA concentrations. Afterwards, variables reaching statistical significance were inserted in a stepwise multivariate linear regression model to define the independent predictors of PWV, AI and AP. Furthermore, HOMA only, and not fasting insulin or glucose levels, was included in the model to avoid possible collinearity.

Finally, a linear regression analysis was performed to test the association between traditional CV risk factors and serum UA with increased arterial stiffness (AS) considered as PWV >10 m/s [15]. Uric acid levels were considered as a continuous variable, and the model was adjusted for established CV risk factors.

The investigation of biological interactions between UA and HOMA that may explain increased stiffness was performed, as previously described [19], by dividing the study population into four groups according to the median values of serum UA and HOMA. Biological association (synergism) between HOMA and serum UA was primarily defined as a deviation from an additive model occurring when the observed odds ratio (OR) for increased vascular stiffness of individuals with both high HOMA and high serum UA was greater than the ones obtained by adding the odds ratios of subjects with high HOMA and low serum UA or low HOMA and high serum UA minus one. The correlation between HOMA and serum UA for increased vascular stiffness was also investigated by assessing the deviation from a multiplicative model (statistical interaction) occurring when the observed OR for subjects with both HOMA and serum UA values above the median was higher than the product between the OR of patients with high HOMA and low serum UA and the OR of patients with low HOMA and high serum UA.

Differences were assumed to be significant for *p* < 0.05. All analyses were carried out with the statistical package SPSS 20.0 for Windows (SPSS Inc., Chicago, IL, USA).

### 2.6. Ethical Approval

All evaluations were made according to the Declaration of Helsinki after obtaining approval by the local Ethical Committee and written informed consent from each subject (code protocol number 2012.63).

## 3. Results

### 3.1. Study Population 

Table 1 reports the biochemical and anthropometric characteristics of the whole cohort and of the subgroups obtained following the stratification of the study patients in three tertiles according to their serum UA levels. There were no significant differences among groups for age, LDL, triglycerides, and prevalence of smokers. Significant differences were, by contrast, observed for gender, BMI, fasting glucose, insulin, HOMA index, HDL, e-GFR and hs-CRP. Specifically, there was a higher prevalence of males in the tertile with the highest UA levels and BMI, fasting glucose and insulin levels, HOMA index and hs-CRP concentration increased progressively from the first to the third tertile, while HDL and e-GFR values progressively decreased. Of interest, male patients showed higher UA values than females (5.3 ± 1.5 versus 5.1 ± 1.6 mg/dL; *p* = 0.022).

### 3.2. Hemodynamic Parameters 

Peripheral and aortic hemodynamic parameters of the whole study population and of the three subgroups are shown in Table 2. There were no significant differences among the three tertiles for heart rate (HR) and DBP. Conversely, the values of SBP and clinical PP, central PAS and central PP, AP, AI and PWV progressively increased, while the values of central DBP progressively decreased from the lowest to the highest serum UA tertiles. 

### 3.3. Correlational Analysis 

The correlation between PWV and several covariates in the whole study population was assessed by linear regression analysis (Table 3). Pulse wave velocity was linearly correlated with serum UA (*r* = 0.453, *p* < 0.0001), HOMA (*r* = 0.391, *p* < 0.0001), hs-CRP (*r* = 0.159, *p* < 0.0001), SBP (*r* = 0.158, *p* < 0.0001), age (*r* = 0.108, *p* < 0.0001) and LDL (*r* = 0.077, *p* = 0.005), while HDL values and UA levels showed an inverse relationship (*r* = −0.084, *p* = 0.003). 

### 3.4. Multivariate Analysis

To identify the independent predictors of PWV, variables showing statistically significant correlations in the linear regression analysis were included in a stepwise multivariate linear regression model, with gender and smoking status as dichotomic values (Table 4). 

Serum UA was the strongest predictor of PWV, justifying 20.5% of its variation. Other predictors were HOMA, SBP, e-GFR, age and hs-CRP, accounting for another 6.3%, 0.9%, 0.7%, 0.5% and 0.3% of its variation, respectively. 

Finally, in the adjusted logistic regression analysis, a 1 mg/dL increase in serum UA values resulted in an 80% increased risk of AS, defined as PWV > 10 m/s (Table 5, OR = 1.80; 95% CI: 1.55–2.08). In this model, the other variables associated with increased AS were HOMA and age. Specifically, a 1 U increase of HOMA and 10 years increase of age were associated, respectively, with an increase of 47% and 26% of risk of AS. 

### 3.5. Interaction Analysis

**Figure 1:**
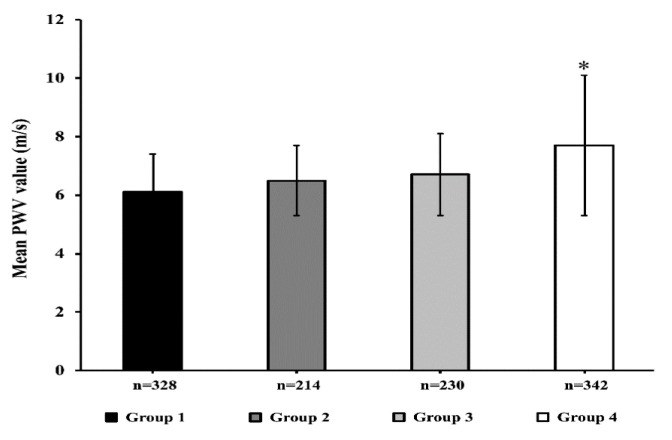
Mean pulse wave velocity (PWV) values in four groups according to the median of HOMA and uric acid (UA). Group 1 = individuals with UA < median values and HOMA < median (*n* = 328, black column); Group 2 = UA > median, HOMA < median (*n* = 214, dark grey column); Group 3 = UA < median, HOMA > median (*n* = 230, light grey column); Group 4 = UA > median, HOMA > median (*n* = 342, white column). * *p* < 0.0001 for the fourth group as compared to the three other groups.

Figure 1 shows the average values of PWV in the sub-groups defined according to the median of HOMA (2.75) and UA levels (4.9 mg/dL). Patients with both HOMA and UA above the median had significantly higher values of PWV as compared to the other groups (*p* < 0.0001).

In the univariate logistic regression analysis, serum UA (OR = 3.69, 95% CI: 2.19–6.22) and HOMA (OR = 2.51, 95% CI: 1.30–4.84) values above the median were both associated with a greater risk of increased AS (PWV > 10 m/s).

**Figure 2:**
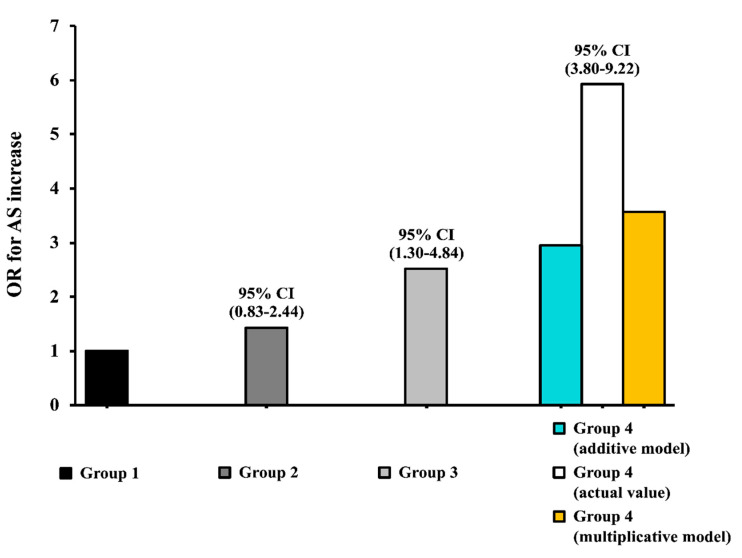
Graphic representation of the biological interaction analysis between serum uric acid (UA) and HOMA on increased arterial stiffness (AS) (PWV > 10 m/s). Group 1 (UA < median values and HOMA < median) represented the reference group (black column); observed values are reported for Group 2 (UA > median, HOMA < median, dark grey column) and Group 3 (UA < median, HOMA > median, light grey column). Observed actual values (white column) and expected values according to an additive (light blue column) or multiplicative (yellow column) model are reported for Group 4 (UA > median, HOMA > median).

A biological interaction analysis performed adjusting for a series of potential confounding factors (age, BMI, DBP, SBP, LDL, HDL, triglycerides, e-GFR and hs-CRP) highlighted a significant interaction between UA and HOMA for increased AS risk. Specifically, the interaction analysis carried out according to an additive model, which represents the preferred model for testing a biological interaction in etiological studies [21,22], showed that the joint effect of UA and HOMA index was significantly greater than the effect that would have been expected in the absence of any interaction. In particular, patients with both HOMA and UA above the median showed an observed OR = 5.92, 95% CI: 3.80–9.22, compared to an expected value of (2.51 (OR for increased AS in patients with HOMA above the median and UA under the median, group 3 in Figure 2) + 1.41 (OR in patients with HOMA under the median and UA above the median, group 2 in Figure 2) − 1 = 2.93) (synergy index: 2.54). This result indicates that the two biomarkers interact synergistically in promoting damage to the arteries in the study population (Figure 2); patients presenting both serum UA and HOMA values above the median had, indeed, a six times greater risk of increased AS (OR = 5.92, 95% CI: 3.80–9.22). The same interaction analysis was performed separately for males and females. In both subgroups, patients presenting serum UA and HOMA values above the median showed a higher risk for increased AS, significantly more elevated than expected (observed OR = 13.3, 95% CI: 3.98–22.9 compared to an expected value of (2.04 + 4.22)− 1 = 5.26 in males; observed OR = 9.90, 95% CI: 3.85–25.4 compared to an expected value of (2.20 + 2.10)− 1 = 3.30 in females), thus showing that HOMA and UA interact synergistically in promoting vascular damage both in males and females, but the effect was more pronounced in male patients.

Notably, when the interaction analysis was conducted according to a multiplicative model, both on univariate and multivariate logistic regression models, the OR observed for vascular damage in subjects with both serum UA and HOMA values above the median was greater than the simple product between the OR in patients with serum UA or HOMA values above the median (observed OR = 5.92, the 95% CI: 3.80–9.22 compared to the expected value of 1.42 × 2.51 = 3.56) (Figure 2).

## 4. Discussion

Several previous studies have provided evidence suggesting a possible correlation between subclinical organ damage and increased serum UA [23,24,25,26,27]. The present findings, obtained in a cohort of 1114 non-diabetic subjects with normal renal function and a novel diagnosis of hypertension, consolidated the available data and shed light on the mechanisms underlying this association.

Our data showed that patients in the highest UA tertile (mean UA serum levels 7.1 ± 1.1 mg/dL) had a worse metabolic and hemodynamic status with significantly higher levels of PWV and its hemodynamic correlates AI and AP. A further proof for the role of UA levels in vascular damage was obtained from the correlational analysis, demonstrating that UA was the major predictor of PWV justifying 20.5% of its variation. Interestingly, from the adjusted logistic regression analysis emerges that an increase of 1 mg/dL in serum UA values results in an almost double risk of AS, defined as PWV > 10 m/s. This observation has an important clinical relevance, because it is demonstrated that AS is an independent predictor of cardiovascular events and mortality from all-cause. Specifically, an increase of 1 m/s of PWV raises by 14% the global risk of CV events, by 15% the risk of cardiovascular mortality, and by another 15% the risk of all-cause mortality [28]. The results reported here are in keeping with those observed by Ding X.H. et al. [29] in a population of normotensive Chinese subjects. Furthermore, our data strengthen evidence from a recent study suggesting that UA serum levels may help in the reclassification of actual CV risk in subjects affected by arterial hypertension [10].

Finally, our study offers, to the best of our knowledge, the first proof of a biological interaction between UA and HOMA index in increasing the risk of AS. Particularly, patients with both UA and HOMA values higher than the median value presented a six times greater risk of PWV > 10 m/s. This finding was observed both in male then in female patients, but the effect was more pronounced in males. This demonstrates a synergistic action between UA and HOMA in inducing subclinical vascular damage, suggesting that IR itself may be one of the major mechanisms trough which UA increases AS in our cohort of newly diagnosed, never-treated hypertensive individuals. The ability, demonstrated in experimental studies, of UA to interfere with insulin signalling [30], with IGF-1 levels [31], with the activations of inflammatory processes and systems, such as RAAS and sympathetic system [32,33], promoting oxidative stress [11] and endothelial dysfunction [12], are all possible mechanisms underlying this association.

The present study has a number of strengths including the size of the cohort analysed, the exclusion of confounding conditions known to affect UA levels, the recruitment of newly diagnosed subjects naive to drugs that may alter UA concentration and the inclusion of childbearing-age women only to account for the established effect of the loss of oestrogen uricosuric action. Nonetheless, the cross-sectional design of our study prevents the demonstration of a causal relationship between higher levels of UA and increased AS, even if previous preclinical [32,33] and clinical studies sustain the biological plausibility of our hypothesis [34,35,36]. Possible mechanisms underlying the interaction between UA and HOMA index and their effects on vascular damage may include increased levels of free oxygen radicals (ROS) and enhanced inflammation, reported both in subjects with insulin resistance and in individuals with hyperuricemia [37]. Moreover, it is known that adiponectin, the most abundant adipose tissue specific protein, has antiatherogenic properties, and decreased levels of this protein are not only correlated with T2DM and insulin sensitivity but have also been observed in hyperuricemic subjects [38]. Future in vitro and/or in vivo studies are needed to address the role of these putative mediators.

Furthermore, the present data have been obtained in Caucasian individuals, whether our findings can be extended to subjects from different ethnic groups remains to be determined. Finally, another important limitation of the study consists in the fact that we have not measured physical activity at baseline, and it is well known that it represents an important factor affecting AS.

In conclusion, the results emerging from our study strengthen the available evidence on the importance of UA as a cause and predictor of cardiovascular damage. Furthermore, our data appear highly clinically relevant since the knowledge of the pathophysiological mechanisms may allow early interventions on vascular damage to prevent clinical events.

## Figures and Tables

**Table 1:** Anthropometric and biochemical characteristics of the study population according to increasing tertiles of serum UA.

	All	I Tertile	II Tertile	III Tertile	*p*
Variables	(*n* = 1114)	(*n* = 371)	(*n* = 371)	(*n* = 372)	
Gender, m/f	634/480	191/180	214/157	229/143	0.020 *
Age, years	48.9 ± 11.5	48.1 ± 10.9	49.3 ± 11.9	48.9 ± 11.5	0.260
BMI, Kg/m^2^	29.5 ± 5.6	29.2 ± 5.8	29.2 ± 5.2	30.1 ± 5.7	0.045
Glucose, mg/dL	92.7 ± 10.3	91.4 ± 9.9	92.1 ± 10.1	94.6 ± 10.7	<0.0001
Insulin, mU/mL	13.2 ± 6.8	11.2 ± 5.3	13.1 ± 6.6	15.6 ± 7.6	<0.0001
HOMA	3.1 ± 1.7	2.5 ± 1.3	2.9 ± 1.6	3.7 ± 1.9	<0.0001
Serum LDL, mg/dL	123.6 ± 34.7	120.4 ± 35.3	125.5 ± 33.1	125.1 ± 35.5	0.085
Serum HDL, mg/dL	50.8 ± 13.4	52.1 ± 13.9	50.8 ± 13.6	49.3 ± 12.7	0.016
Serum Triglycerides, mg/dL	127.3 ± 57.6	123.7 ± 58.1	130.3 ± 58.2	127.9 ± 56.6	0.289
e-GFR, mL/min/1.73 m^2^	106.2 ± 28.2	110.1 ± 31.6	105.4 ± 26.6	103.3 ± 25.8	0.005
hs-CRP mg/dL	3.0 ± 2.1	2.8 ± 1.9	2.9 ± 2.1	3.4 ± 2.4	<0.0001
Smokers, (*n*/%)	310/27.8%	95/25.6%	104/28.0%	111/29.8%	0.434 *

Values data are mean ± SD; * χ^2^ test. Abbreviations: BMI, body max index; HOMA, homeostatic model assessment; e-GFR, estimated glomerular filtration rate; hs-CRP, high-sensitivity C-reactive protein.

**Table 2:** Peripheral and central hemodynamic parameters of the study population, according to increasing tertiles of serum UA.

	All	I Tertile	II Tertile	III Tertile	*p*
Variables	(*n* = 1114)	(*n* = 371)	(*n* = 371)	(*n* = 372)	
HR, bpm	67.5 ± 20.9	67.5 ± 11.5	67.1 ± 12.5	68.7 ± 33.1	0.591
SBP, mmHg	143.9 ± 14.1	142.4 ± 13.5	143.4 ± 13.9	145.9 ± 14.3	0.002
DBP, mmHg	89.3 ± 8.2	90,1 ± 8,2	88.6 ± 8.1	87.9 ± 9.3	0.064
PP, mmHg	54 ± 15.4	53 ± 13.5	53 ± 15.2	56 ± 15.6	0.004
Central-SBP, mmHg	131 ± 13.4	129 ± 12.9	131 ± 14.5	132 ± 12.7	0.025
Central-DBP, mmHg	88 ± 9.4	89 ± 9.2	88 ± 9.4	87 ± 9.3	0.008
Central-PP, mmHg	42 ± 14.3	40 ± 14.8	42 ± 14.1	44 ± 13.6	<0.0001
AP, mmHg	11.1 ± 7.1	9.1 ± 6.2	10.8 ± 6.7	13.2 ± 7.5	<0.0001
AI, %	25.6 ± 12.9	22.1 ± 12.3	25.4 ± 12.9	29.2 ± 12.7	<0.0001
PWV, m/s	6.8 ± 1.9	6.2 ± 1.3	6.4 ± 1.1	7.8 ± 2.5	<0.0001

Values data are mean ± SD. Abbreviations: HR, heart rate; SBP, systolic blood pressure; DBP, diastolic blood pressure; PP, pulse pressure; AP, augmentation pressure; AI, augmentation index; PWV, pulse wave velocity.

**Table 3:** Linear regression analysis on pulse wave velocity (PWV) as a dependent variable in the whole study population.

	PWV	
	*R*	*p*
Age, years	0.108	<0.0001
BMI, Kg/m^2^	0.025	0.206
SBP, mmHg	0.158	<0.0001
DBP, mmHg	−0.048	0.055
Serum HDL, mg/dL	−0.084	0.003
Serum LDL, mg/dL	0.077	0.005
Serum Triglycerides, mg/dL	0.013	0.330
e-GFR, mL/min/1.73 m^2^	−0.024	0.212
hs-CRP, mg/dL	0.159	<0.0001
HOMA	0.391	<0.0001
Uric Acid, mg/dL	0.453	<0.0001

Values data are mean ± SD. Abbreviations: BMI, body max index; SBP, systolic blood pressure; DBP, diastolic blood pressure; e-GFR, estimated glomerular filtration rate; hs-CRP, high-sensitivity C-reactive protein; HOMA, homeostatic model assessment.

**Table 4:** Multivariate stepwise regression analysis on PWV as dependent variable in whole study population.

	*R* ^2^	*p*
Uric Acid, mg/dL	20.5	<0.0001
HOMA	6.3	<0.0001
SBP, mmHg	0.9	<0.0001
Age, years	0.5	0.008
e-GFR mL/min/1.73 m^2^	0.7	0.001
hs-CRP, mg/dL	0.3	0.045

Values data are mean ± SD. Abbreviations: PWV, pulse wave velocity; HOMA, homeostatic model assessment; SBP, systolic blood pressure; e-GFR, estimated glomerular filtration rate; hs-CRP, high-sensitivity C-reactive protein.

**Table 5:** Logistic regression analysis on vascular damage (PWV > 10 m/s).

	OR	95% CI
Uric Acid, 1 mg/dL	1.80	1.55–2.08
Homa, 1 U	1.47	1.29–1.66
Age, 10 years	1.26	1.01–1.59

Values data are mean ± SD. Abbreviations: HOMA, homeostatic model assessment.

## References

[1] So A., Thorens B. (2010). Uric acid transport and disease. J. Clin. Investig..

[2] Perticone F., Sciacqua A., Perticone M., Arturi F., Scarpino P.E., Quero M., Sesti G. (2012). Serum uric acid and 1-h postload glucose in essential hypertension. Diabetes Care.

[3] Meshkani R., Zargari M., Larijani B. (2011). The relationship between uric acid and metabolic syndrome in normal glucose tolerance and normal fasting glucose subjects. Acta Diabetol..

[4] Bonora E., Targher G., Zenere M.B., Saggiani F., Cacciatori V., Tosi F., Travia D., Zenti M.G., Branzi P., Santi L. (1996). Relationship of uric acid concentration to cardiovascular risk factors in young men. Role of obesity and central fat distribution. The Verona Young Men Atherosclerosis Risk Factors Study. Int. J. Obes..

[5] Perticone F., Maio R., Tassone J.E., Perticone M., Pascale A., Sciacqua A., Sesti G. (2013). Interaction between uric acid and endothelial dysfunction predicts new onset of diabetes in hypertensive patients. Int. J. Cardiol..

[6] Dehghan A., Van Hoek M., Sijbrands E.J.G., Hofman A., Witteman J.C.M. (2008). High serum uric acid as a novel risk factor for type 2 diabetes. Diabetes Care.

[7] Krishnan E., Pandya B.J., Chung L., Hariri A., Dabbous O. (2012). Hyperuricemia in young adults and risk of insulin resistance, prediabetes, and diabetes: A 15-year follow-up study. Am. J. Epidemiol..

[8] Verdecchia P., Schillaci G., Reboldi G.P., Santeusanio F., Porcellati C., Brunetti P. (2000). Relation between serum uric acid and risk of cardiovascular disease in essential hypertension: The PIUMA study. Hypertension.

[9] Niskanen L.K., Laaksonen D.E., Nyyssönen K., Alfthan G., Lakka H.M., Lakka T.A., Salonen J.T. (2004). Uric acid level as a risk factor for cardiovascular and all-cause mortality in middle-aged men: A prospective cohort study. Arch. Intern. Med..

[10] Perticone M., Tripepi G., Maio R., Cimellaro A., Addesi D., Baggetta R., Sciacqua A., Sesti G., Perticone F. (2017). Risk reclassification ability of uric acid for cardiovascular outcomes in essential hypertension. Int. J. Cardiol..

[11] Sautin Y.Y., Nakagawa T., Zharikov S., Johnson R.J. (2007). Adverse effects of the classic antioxidant uric acid in adipocytes: NADPH oxidase-mediated oxidative/nitrosative stress. Am. J. Physiol.-Cell Physiol..

[12] Zoccali C., Maio R., Mallamaci F., Sesti G., Perticone F. (2006). Uric acid and endothelial dysfunction in essential hypertension. J. Am. Soc. Nephrol..

[13] Fiorentino T.V., Sesti F., Succurro E., Pedace E., Andreozzi F., Sciacqua A., Hribal M.L., Perticone F., Sesti G. (2018). Higher serum levels of uric acid are associated with a reduced insulin clearance in non-diabetic individuals. Acta Diabetol..

[14] Andreozzi F., Succurro E., Mancuso M.R., Perticone M., Sciacqua A., Perticone F., Sesti G. (2007). Metabolic and cardiovascular risk factors in subjects with impaired fasting glucose: The 100 versus 110 mg/dL threshold. Diabetes Metab. Res. Rev..

[15] Williams B., Mancia G., Spiering W., Agabiti Rosei E., Azizi M., Burnier M., Clement D., Coca A., De Simone G., Dominiczak A. (2018). 2018 ESC/ESH Guidelines for the Management of Arterial Hypertension: The Task Force for the Management of Arterial Hypertension of the European Society of Cardiology and the European Society of Hypertension: The Task Force for the Management of Arterial Hypertension of the European Society of Cardiology and the European Society of Hypertension. J. Hypertens..

[16] Levey A.S., Stevens L.A., Schmid C.H., Zhang Y., Castro A.F., Feldman H.I., Kusek J.W., Eggers P., Van Lente F., Greene T. (2009). A new equation to estimate glomerular filtration rate. Ann. Intern. Med..

[17] Perticone M., Maio R., Tassone E.J., Tripepi G., Di Cello S., Miceli S., Caroleo B., Sciacqua A., Licata A., Sesti G. (2015). Insulin-resistance HCV infection-related affects vascular stiffness in normotensives. Atherosclerosis.

[18] Chen C.H., Ting C.T., Nussbacher A., Nevo E., Kass D.A., Pak P., Wang S.P., Chang M.S., Yin F.C.P. (1996). Validation of carotid artery tonometry as a means of estimating augmentation index of ascending aortic pressure. Hypertension.

[19] Covic A., Siriopol D. (2015). Pulse wave velocity ratio: The new “gold standard” for measuring arterial stiffness. Hypertension.

[20] Tanaka A., Tomiyama H., Maruhashi T., Matsuzawa Y., Miyoshi T., Kabutoya T. (2018). Physiological diagnostic criteria for vascular failure. Hypertension.

[21] Rothman K.J., Greenland S., Walker A.M. (1980). Concepts of interaction. Am. J. Epidemiol..

[22] De Mutsert R., Jager K.J., Zoccali C., Dekker F.W. (2009). The effect of joint exposures: Examining the presence of interaction. Kidney Int..

[23] Ramirez A.J., Christen A.I., Sanchez R.A. (2018). Serum Uric Acid Elevation Is Associated To Arterial Stiffness In Hypertensive Patients With Metabolic Disturbances. Curr. Hypertens. Rev..

[24] Canepa M., Viazzi F., Strait J.B., Ameri P., Pontremoli R., Brunelli C., Studenski S., Ferrucci L., Lakatta E.G., Alghatrif M. (2017). Longitudinal Association between Serum Uric Acid and Arterial Stiffness: Results from the Baltimore Longitudinal Study of Aging. Hypertension.

[25] Vlachopoulos C., Xaplanteris P., Vyssoulis G., Bratsas A., Baou K., Tzamou V., Aznaouridis K., Dima I., Lazaros G., Stefanadis C. (2011). Association of serum uric acid level with aortic stiffness and arterial wave reflections in newly diagnosed, never-treated hypertension. Am. J. Hypertens..

[26] Mulè G., Riccobene R., Castiglia A., D’Ignoto F., Ajello E., Geraci G., Guarino L., Nardi E., Vaccaro F., Cerasola G. (2014). Relationships between mild hyperuricaemia and aortic stiffness in untreated hypertensive patients. Nutr. Metab. Cardiovasc. Dis..

[27] Nagano S., Takahashi M., Miyai N., Oka M., Utsumi M., Shiba M., Mure K., Takeshita T., Arita M. (2017). Association of serum uric acid with subsequent arterial stiffness and renal function in normotensive subjects. Hypertens. Res..

[28] Vlachopoulos C., Aznaouridis K., Stefanadis C. (2010). Prediction of Cardiovascular Events and All-Cause Mortality With Arterial Stiffness. A Systematic Review and Meta-Analysis. J. Am. Coll. Cardiol..

[29] Ding X.H., Wang X., Cao R., Yang X., Xiao W., Zhang Y., Bai Y., Wu H., Ye P. (2017). A higher baseline plasma uric acid level is an independent predictor of arterial stiffness a community-based prospective study. Medicine (United States).

[30] Tassone E.J., Cimellaro A., Perticone M., Hribal M.L., Sciacqua A., Andreozzi F., Sesti G., Perticone F. (2018). Uric acid impairs insulin signaling by promoting ENPP1 binding to insulin receptor in human umbilical vein endothelial cells. Front. Endocrinol. (Lausanne).

[31] Sesti G., Hribal M.L., Procopio T., Fiorentino T.V., Sciacqua A., Andreozzi F., Marini M.A., Perticone F. (2014). Low circulating insulin-like growth factor-1 levels are associated with high serum uric acid in nondiabetic adult subjects. Nutr. Metab. Cardiovasc. Dis..

[32] Mazzali M., Kanellis J., Han L., Feng L., Xia Y.Y., Chen Q., Kang D.H., Gordon K.L., Watanabe S., Nakagawa T. (2002). Hyperuricemia induces a primary renal arteriolopathy in rats by a blood pressure-independent mechanism. Am. J. Physiol.-Ren. Physiol..

[33] Mazzali M., Hughes J., Kim Y.G., Jefferson J.A., Kang D.H., Gordon K.L., Lan H.Y., Kivlighn S., Johnson R.J. (2001). Elevated uric acid increases blood pressure in the rat by a novel crystal-independent mechanism. Hypertension.

[34] Hsu P.F., Chuang S.Y., Cheng H.M., Sung S.H., Ting C.T., Lakatta E.G., Yin F.C.P., Chou P., Chen C.H. (2013). Associations of serum uric acid levels with arterial wave reflections and central systolic blood pressure. Int. J. Cardiol..

[35] Ishizaka N., Ishizaka Y., Toda E.I., Hashimoto H., Nagai R., Yamakado M. (2007). Higher serum uric acid is associated with increased arterial stiffness in Japanese individuals. Atherosclerosis.

[36] Ng K.P., Stringer S.J., Jesky M.D., Yadav P., Athwal R., Dutton M., Ferro C.J., Cockwell P. (2014). Allopurinol is an independent determinant of improved arterial stiffness in chronic kidney disease: A cross-sectional study. PLoS ONE.

[37] Doehner W., Jankowska E.A., Springer J., Lainscak M., Anker S.D. (2016). Uric acid and xanthine oxidase in heart failure—Emerging data and therapeutic implications. Int. J. Cardiol..

[38] Nishizawa T., Taniura T., Nomura S. (2015). Effects of febuxostat on platelet-derived microparticles and adiponectin in patients with hyperuricema. Blood Coagul. Fibrinolysis.

